# Binding of Quercetin Derivatives toward G-Tetrads as Studied by the Survival Yield Method

**DOI:** 10.1021/acsomega.3c06016

**Published:** 2023-10-11

**Authors:** Olga Stężycka, Magdalena Frańska

**Affiliations:** Institute of Chemistry and Technical Electrochemistry, Poznań University of Technology, Berdychowo 4, 60-965 Poznań, Poland

## Abstract

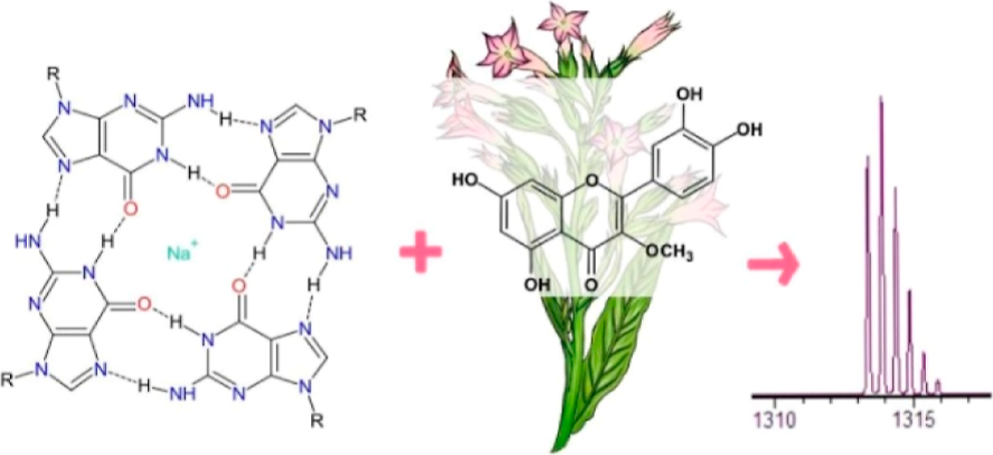

Recently, much interest has been devoted to finding effective G-quadruplex ligands, both of synthetic or natural origins, which may be of potential use in the field of cancer therapy. Among compounds of natural origin, a common flavonol quercetin has attracted notable attention. Yet, only a modest number of papers have been concerned with a comparison of quercetin conjugates binding to G-quadruplexes. In this study, we applied the survival yield (SY) method in order to compare the stability of G-tetrad complexes with quercetin and its conjugates, namely, 3-O-glycosides and O-methylated conjugates. According to the determined values of *E*_comδ50_, flavonol glycosides bind most effectively with G-tetrads, whereas, among flavonols, 3-O-methylquercetin makes the most effective bonds. Because the aglycone structure is of crucial importance for biological processes, 3-O-methylquercetin seems to be a suitable candidate for anticancer therapeutics, and the extracts from the plants, which contain high amounts of 3-O-methylquercetin or its glycosides, should be considered as interesting materials for preparation of pharmaceuticals or dietary supplements.

## Introduction

Flavonoids make up a large group of polyphenolic compounds which occur naturally and constitute a significant part of the human diet, e.g., they are abundant in fruits, vegetables, seeds, wine, and tea. An exemplary compound that belongs to this group is quercetin—a flavonol which is known for its health-promoting properties, e.g., antioxidant, anti-inflammatory, antibacterial, and anticancer effects.^1−3^ Quercetin, often occurs naturally in the form of glycosides, usually glycosylation takes place at the 3-O position, and the most common glycosylated quercetin conjugates are rutin (quercetin-3-O-rutinoside), quercitrin (quercetin 3-O-rhamnoside), guaijaverin (quercetin 3-O-arabinoside), hyperoside (quercetin-3-O-galactoside), and isoquercetin (quercetin-3-O-glucoside). Quercetin glycosides are also of special interest with respect to human health. For example, rutin is a common component of pharmaceuticals and dietary supplements^4−7^ and isoquercetin can be used as an adjunct therapy agent during cancer treatment.^8,9^ Other interesting quercetin derivatives are O-methylated conjugates, from which the most common are rhamnetin (7-O-methylquercetin) and isorhamnetin (3′-O-methylquercetin), which also may have a high value of development and application due to their pharmacological activities.^10,11^ Other O-methylated quercetin conjugates are relatively rare but are also interesting with respect to their potential application, e.g., the presence of 3-O-methylquercetin in the leaves of *Nicotiana tabacum* L. may be interesting because there is a strong socioeconomic interest in finding an alternative use for tobacco.^12^

**Figure 1:**
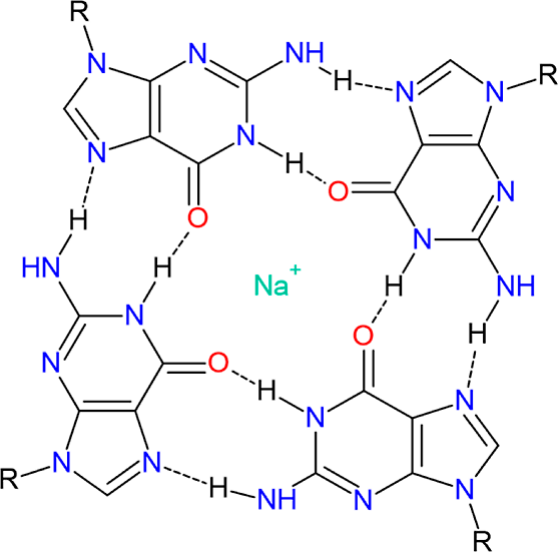
Structure of G-tetrad; R stands for ribose or deoxyribose.

Nucleic acid fragments rich in guanosine have a characteristic spatial structure with important biological properties. Four interacting guanosines form a G-tetrad. This structure is the basic building block of the G-quadruplex, which is stabilized by metal ions, e.g., Na^+^ and K^+^ (Figure 1).

These structures are found in telomeres and regulatory sites in cells. The presence of G-quadruplexes at these sites in cells suggests that G-quadruplexes may play a role in the regulation of gene transcription. Structure, occurrence, and function have made G-quadruplexes a promising target in the search for new therapies and drugs. Recently, there is growing interest to design molecules that are able to interact with G-quadruplexes, which may be of importance toward cancer therapy^13,14^ and antiviral therapy^15^ or to design G-quadruplex probes.^16^ It gives motivation to develop computational and physicochemical methods for searching for effective G-quadruplex ligands.^17−19^ The ligands can be both synthetic or of natural origin.^20,21^ Among the latter ligands, quercetin has attracted notable attention.^22^ Quercetin binding to G-quadruplexes has been studied by spectroscopic methods (e.g., NMR, circular dichroism), calorimetric titration, molecular docking, and density functional theory.^23−28^ On the other hand, quercetin conjugates binding to G-quadruplexes has not been widely studied. Wang et al. have studied G-quadruplex interactions with isorhamnetin-3-O-neohesperidoside by using capillary electrophoresis and electrospray ionization mass spectrometry (ESI-MS),^29^ Sun et al. have studied the G-quadruplex–rutin interaction by ^1^H NMR spectroscopy.^30^ To the best of our knowledge, there are only two literature reports that contain a comparison between quercetin and its glycoside rutin binding with G-quadruplexes. Ribaudo et al. have found, by using collision-induced dissociation mass spectrometry (CID-MS) and molecular docking, that the G-quadruplex–rutin complex is more stable than that of G-quadruplex–quercetin.^31^ On the other hand, the spectroscopic results obtained by Tawani and Kumar suggest quercetin-bound G-quadruplexes to be more effective than rutin.^32^

**Figure 2:**
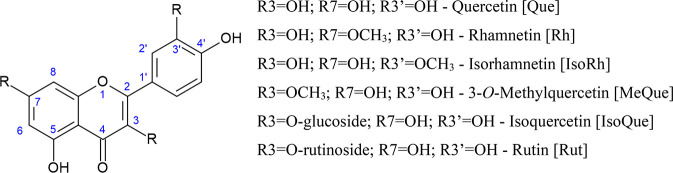
Structures of quercetin and its conjugates studied in this work.

In this paper, we applied the survival yield (SY) method in order to compare the stability of G-tetrad complexes with quercetin and its conjugates, two 3-O-glycosides and three O-methylated conjugates (Figure 2).

## Materials and Methods

The flavonols, flavonol glycosides, and nucleosides were obtained from Sigma-Aldrich (Poznań, Poland).

The electrospray ionization–collision-induced dissociation-tandem mass spectrometry (ESI-CID-MS/MS) analyses of the adducts of interest were performed on a Waters/Micromass Q-TOF Premier mass spectrometer (software MassLynx v.4.1, Manchester, UK). Methanol solutions containing guanosine (G_r_) or deoxyguanosine (G_dr_) and flavonol (concentrations ∼ 10^–4^ and 5×10^–5^ M, respectively) were infused into the ESI source by a syringe pump at a flow rate of 5 μL/min. The electrospray voltage was 2.7 kV and the cone voltage was 30 V. The source temperature was 80 °C and the desolvation temperature was 250 °C. Nitrogen was used as the cone gas and desolvating gas at the flow rates of 0.8 and 13 L/min, respectively. Argon was used as a collision gas (*m*_g_ = 40) at a flow rate of 0.5 mL/min in the T-wave collision cell. This flow rate resulted in a collision cell pressure of 0.3 Pa. The applied collision energy (CE, laboratory frame), the most important parameter for collision-induced dissociation tandem mass spectrometry experiments (CID-MS/MS), is indicated in each of the product ion spectra shown (Supporting Information). It has been already established that under ESI-MS conditions guanosine and deoxyguanosine form G-tetrads (analogously to the biological G-quadruplex), which may be stabilized by various metal cations.^33−35^

The survival yield of the precursor ion subjected to the collision-induced dissociation is defined as
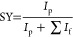
where *I*_p_ stands for the intensity of the peak of precursor ion and *I*_f_ for the intensities of the peaks of fragment ions. Collision energy expressed in terms of the center-of-mass *E*_comδ_ (the maximum kinetic energy available for transfer into internal energy) is defined as
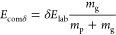
where *E*_lab_ stands for laboratory collision energy, *m*_g_ stands for the mass of the neutral target gas, *m*_p_ stands for the mass of precursor ion, and δ is the correction factor, which reflects the number of degrees of freedom (DOF = 3*N* – 6, *N* stands for the number of atoms in the analyzed ions).^36−38^ The ion [4G_dr_ + Na + Que]^+^ is the smallest from among the analyzed ions, this ion was selected as a reference (DOF_ref_ = 477), so for this ion δ = 1, and for the other ones δ = DOF_ref_/DOF_ion_. The plot of the SY against *E*_comδ_ gives the curve of the precursor ion fragmentation. The value of SY equal to 1/2 permits the determination of *E*_comδ50_, which is the energy required for 50% dissociation of the precursor ion. *E*_comδ50_ can be regarded as a characteristic of the analyzed precursor ion and can be used to measure the relative dissociation energies of the ions of interest.

## Results and Discussion

Table 1 summarizes the detected adducts between G-tetrads (guanosine tetrad [4G_r_ + Na]^+^ at *m*/*z* 1155, or deoxyguanosine tetrad [4G_dr_ + Na]^+^ at *m*/*z* 1091) and flavonol/flavonol glycoside molecules that are the precursor ions subjected to the CID-MS/MS experiments. The precursor ions can be classified as (i) singly charged adducts of 1:1 stoichiometry (e.g., [4G_dr_ + Na + Que]^+^), (ii) doubly charged adducts of 2:2 stoichiometry (e.g., [(4G_r_ + Na + Que)_2_]^2+^), and (iii) doubly charged adducts of 2:1 stoichiometry (e.g., [(4G_dr_ + Na)_2_+Que]^2+^). Exemplary mass spectra are shown in the Supporting Information. Although the adducts of 1:1 and 2:2 stoichiometries are characterized by the same *m*/*z* ratio, they can be differentiated on the basis of the characteristic isotope distribution. The observed isotope distribution indicated that, usually, we dealt with both 1:1 and 2:2 adducts; in other words, the peaks of 1:1 and 2:2 adducts overlap (see Supporting Information, Figure S3). The 1:1 and 2:2 adducts have also different fragmentation pathways, namely, for the former the only product ions are [4G + Na]^+^, whereas for the latter there are two types of product ions, namely, [4G + Na]^+^ and [(4G + Na)_2_ + M]^2+^ (M stands for flavonol/flavonol glycoside molecules). The absence (or very low abundance) or presence of the product ions [(4G + Na)_2_ + M]^2+^ enabled the classification of the adduct as 1:1 or 2:2 ones, respectively (see Supporting Information, Figure S4).

**Table 1:** Detected Adducts between G-Tetrads and Flavonol/Flavonol Glycoside Molecules^a^

ion	*m*/*z*	ion	*m*/*z*
[4G_dr_ + Na + Que]^+^	1393	[4G_dr_ + Na + IsoQue]^+^	1555
[(4G_r_ + Na + Que)_2_]^2+^	1457	[4G_r_ + Na + IsoQue]^+^	1619
[(4G_r_ + Na)_2_ + Que]^2+^	1306	[(4G_dr_ + Na)_2_ + IsoQue]^2+^	1323
[4G_dr_ + Na + Rh]^+^[4G_dr_ + Na + IsoRh]^+^[4G_dr_ + Na + MeQue]^+^	1407	[(4G_r_ + Na)_2_ + IsoQue]^2+^	1387
[(4G_dr_ + Na)_2_ + Rh]^2+^[(4G_dr_ + Na)_2_ + MeQue]^2+^	1249	[(4G_dr_ + Na)_2_ + Rut]^2+^	1396
[(4G_r_ + Na)_2_ + Rh]^2+^[(4G_r_ + Na)_2_ + IsoRh]^2+^[(4G_r_ + Na)_2_ + MeQue]^2+^	1313	[(4G_r_ + Na)_2_ + Rut]^2+^	1460
[(4G_r_ + Na + Rh)_2_]^2+^[(4G_r_ + Na + IsoRh)_2_]^2+^[(4G_r_ + Na + MeQue)_2_]^2+^	1471	[(4G_dr_ + Na + Rut)_2_]^2+^	1701
		[(4G_r_ + Na + Rut)_2_]^2+^	1765

a The *m*/*z* were rounded to the nominal values.

Quercetin and its methylated conjugates prefer formation of 1:1 adducts with deoxyguanosine tetrads and formation of 2:2 adducts with a guanosine tetrad, isoquercetin prefers formation of 1:1 adducts with both tetrads, and rutin prefers formation of 2:2 adducts with both tetrads (Table 1). The obtained plots of SY against *E*_comδ_ for G-tetrad-M adducts are shown in the Supporting Information (Figures S7 and S8).

**Figure 3:**
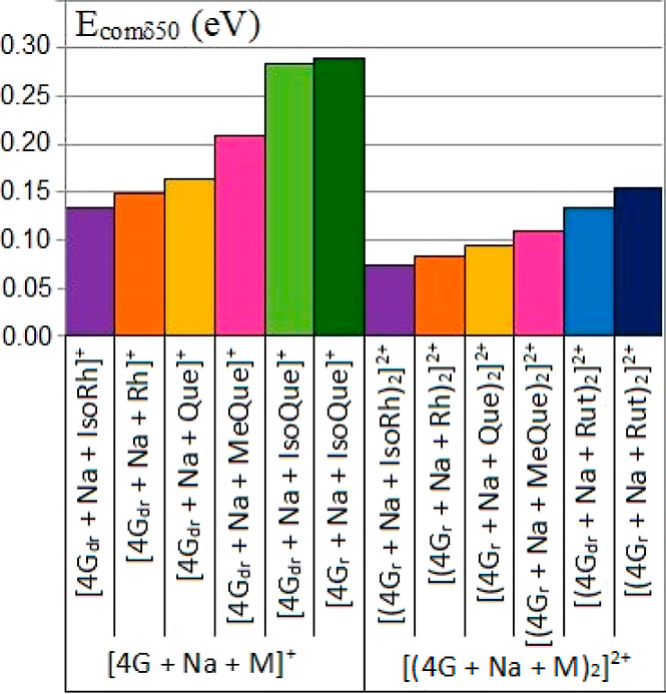
Determined *E*_comδ50_ of 1:1 and 2:2 adducts.

The determined *E*_comδ50_; thus, the energy required for fragmentation of 50% of selected precursor ions, of analyzed adducts enabled rating of the relative gas-phase interaction strength between the G-tetrads and flavonol/flavonol glycosides in the order IsoQue > MeQue > Que > Rh > IsoRh for 1:1 adducts and Rut > MeQue > Que > Rh > IsoRh for 2:2 adducts (Figure 3).

It is evident that glycosylation increases the stability of the analyzed adducts because the sugar moiety is a source of hydrogen bonds.^38−40^ Although G-tetrads are very simple models of G-quadruplexes, (e.g., G-tetrads do not reflect the crucial feature of RNA/DNA topology), the higher stability of G-tetrad–rutin complexes over the stability of G-tetrad–quercetin complexes is analogous to that found for G-quadruplex complexes by Ribaudo et al.^31^ It was also found that rutin may decompose some kinds of G-quadruplexes.^30^ In the product ion spectra of G-tetrad-rutin adducts, the signals of [2G + Na]^+^ and [Rut + Na]^+^ were clearly seen (e.g., Figure S4, *m*/*z* 589 and 633, respectively), which suggests the partial decomposition of G tetrads. We found that rutin has a much higher affinity toward sodium cations than guanosine/deoxyguanosine, in contrast to isoquercetin, as shown in Supporting Information, Figure S6. The high Na^+^ affinity of rutin is caused by the Na^+^ complexation by the bulky sugar moiety; gas-phase decomposition of [Rut + Na]^+^ consists of the loss of a neutral quercetin molecule, whereas gas-phase decomposition [IsoQue + Na]^+^ consists of the loss of a sugar moiety.^41,42^ Because Na^+^ stabilizes the G-quadruplex, complexation of Na^+^ by rutin may also cause G-quadruplex decomposition or, potentially, to be used for recognition of some kinds of G-quadruplex.^30^

The lowest stability was noted for the adduct G-tetrad-IsoRh (Figure 3). There are published examples which indicate that 3′-OH or 4′-OH can form hydrogen bonds with nucleic acids increasing the adduct stabilities.^23,25,43^ Pradhan et al. have found that the presence of catechol moiety (thus the presence of 3′-OH and 4’-OH) may be of crucial importance for the adduct stability.^44^ Therefore, it is clear why isorhamnetin, which has methylated 3′OH, has the lowest stability among the analyzed adducts. 7-OH of quercetin may also play a role in the interactions with nucleic acids;^25^ therefore, rhamnetin, which has methylated 7-OH, also forms less stable adducts in comparison to the quercetin adducts (Figure 3). Surprisingly, 3-O-methylquercetin forms more stable adducts with G-tetrads than quercetin. In other words, the methylation of 3-OH substantially increases the flavonol affinity toward G-tetrads. If there is a free 3-OH group, the C4=O···HO–C3 hydrogen bond can exist (which interferes with C4=O···HO–C5).^25,26^ Methylation of 3-OH yields C4=O, making them more available to form hydrogen bonds with G-tetrads, which may increase the adduct stabilities, similarly, as observed for isoflavone glycosides.^38^

**Figure 4:**
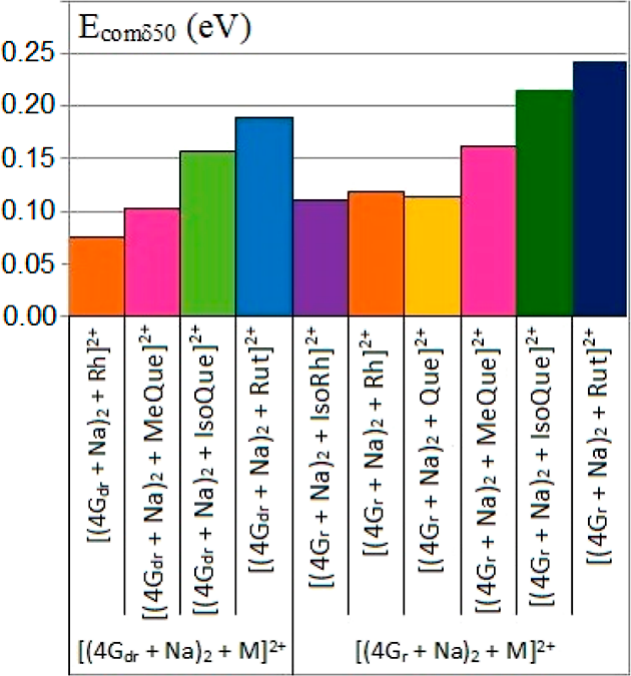
Determined *E*_comδ50_ of 2:1 adducts.

Guanosine tetrad yielded 2:1 adducts with all M molecules, whereas deoxyguanosine tetrad did not yield 2:1 adducts with quercetin and isorhamnetin. The determined values of *E*_comδ50_ of 2:1 adducts are shown in Figure 4.

The adducts [(4G_r_ + Na)_2_-M]^2+^ are definitely more stable than the ones [(4G_dr_ + Na)_2_-M]^2+^, most probably because the additional hydroxyl group in the ribose moiety increases the G-tetrad-M interactions (as a result of additional H-bonds). The highest gas-phase stability of 2:1 adducts was observed for rutin adducts, followed by isoquercetin adducts and then for 3-O-methylquercetin adducts (Figure 4). The 2:1 adducts of quercetin, rhamnetin, and isorhamnetin were characterized by low gas-phase stabilities; although among these three adducts, slightly higher stabilities of rhamnetin adducts may be noted, which is difficult to rationalize. Therefore, for 2:1 adducts, the relative gas-phase interaction strength between the G-tetrads and flavonol/flavonol glycosides increases in the order Rut > IsoQue > MeQue > Rh ∼ Que ∼ IsoRh.

## Conclusions

It was demonstrated that quercetin and its conjugates form adducts with guanosine and deoxyguanosine tetrads under ESI-MS conditions; thus, formation of the adducts between quercetin/quercetin conjugates with G-quadruplexes may be of importance for biological processes. The survival yield method was successfully used to determine the relative gas-phase stabilities of the G-tetrads-flavonol/flavonol glycoside adducts. Although the experiment was performed in the gas phase, thus, under the conditions substantially different from physiological conditions, it is known that studies of gas-phase processes may also have biological implications.^45−47^ According to the determined values of *E*_comδ50_, the flavonol glycosides bind most effectively with G-tetrads. Among the flavonols, 3-O-methylquercetin has the most effective bonds. Although it is a matter of discussion on how the glycosylation of flavonoids affects their bioactivity/bioavailability,^48−50^ it is undisputable that the aglycone structure is of crucial importance for biological processes. Therefore, among the quercetin conjugates, 3-O-methylquercetin seems to be a suitable candidate for anticancer therapeutics and extracts from plants which contain high amounts of 3-O-methylquercetin or its glycosides, e.g., *N. tabacum*, *Ophioglossum pedunculosum*, *Achyrocline satureioides*, and *Rhamnus nakaharai*,^12,51−54^ should be considered as interesting materials for preparation of pharmaceuticals or dietary supplements.

## Supporting Information Available

Full scan ESI mass spectra, product ion spectra, isotope patterns, and plots of SY against *E*_comδ_ (PDF)
