# Supplementary material for: Binding of Quercetin Derivatives toward G-Tetrads as Studied by the Survival Yield Method
Source: ACS Omega. 2023 Oct 11;8(42):39816–21. doi: 10.1021/acsomega.3c06016 (PMC10600882; doi:10.1021/acsomega.3c06016)
Supplement: Supplementary file 1 — ao3c06016_si_001.pdf [file ao3c06016_si_001.pdf]

Supporting Information

Binding of Quercetin Derivatives Toward G-tetrads as Studied by Survival Yield Method

Olga Stężycka and Magdalena Frańska*

Institute of Chemistry and Technical Electrochemistry, Poznań University of Technology, Berdychowo 4, 60-965 Poznań, Poland; E-mail: magdalena.franska@put.poznan.pl

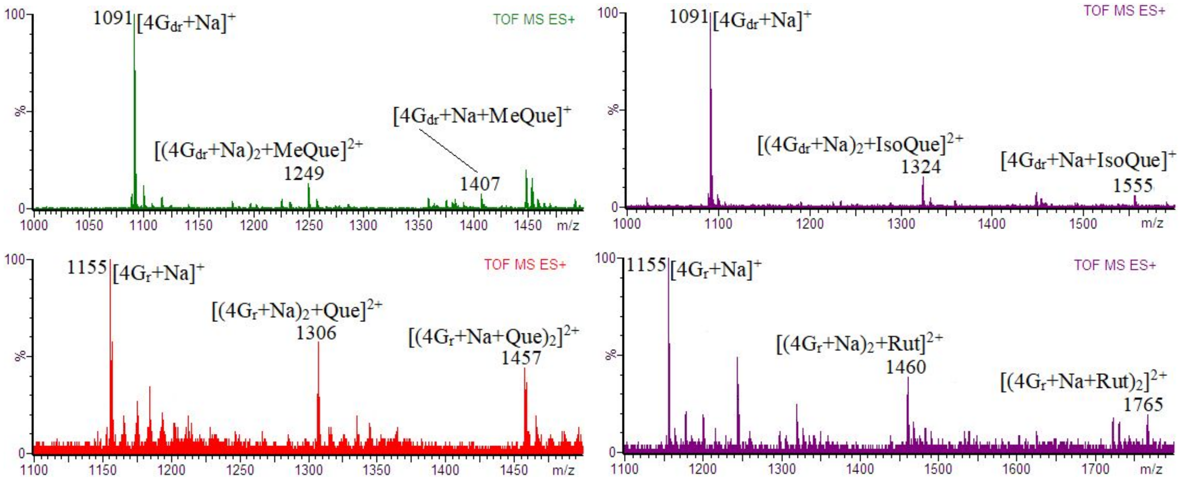

Figure S1. Exemplary full scan ESI mass spectra in the *m/z* range of interest (the *m/z* were rounded to the nominal values).

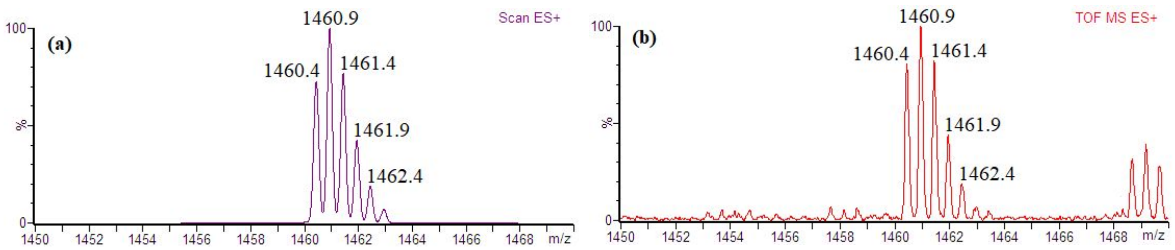

Figure S2. Calculated isotope patterns of $[(4G_r{+}Na)_2{+}Rut]^{2+}$ ($C_{107}H_{134}N_{40}O_{56}Na_2$) - (**a**), isotope patterns obtained - (**b**).

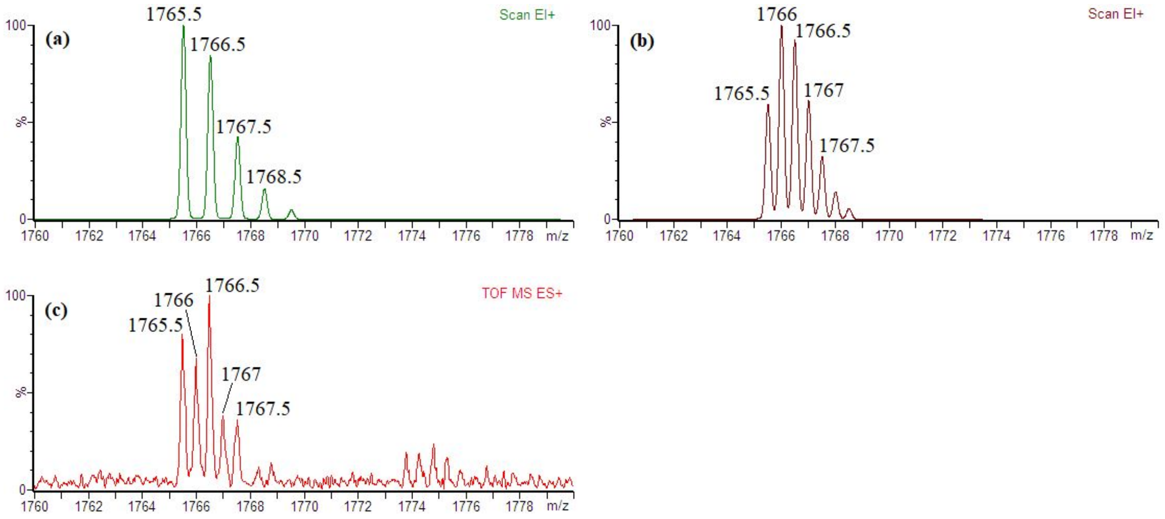

Figure S3. Calculated isotope patterns of $[4G_r{+}Na{+}Rut]^{+}$ ($C_{67}H_{82}N_{20}O_{36}Na$) - (**a**), $[(4G_r{+}Na{+}Rut)_2]^{2+}$ ($C_{134}H_{164}N_{40}O_{72}Na_2$) - (**b**), isotope patterns obtained - **(c)**.

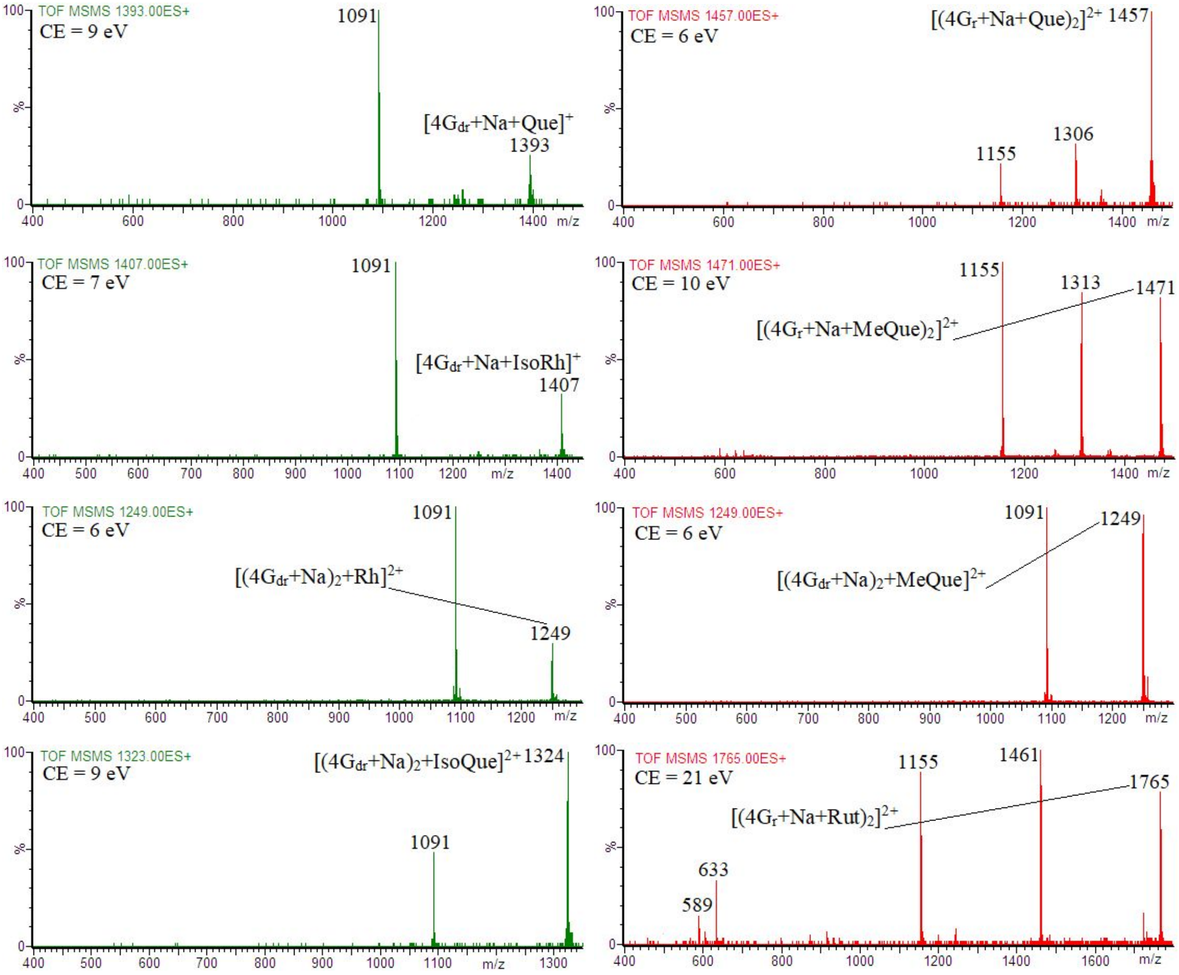

Figure S4. Exemplary product ion spectra of the analyzed adducts (the *m/z* were rounded to the nominal values).

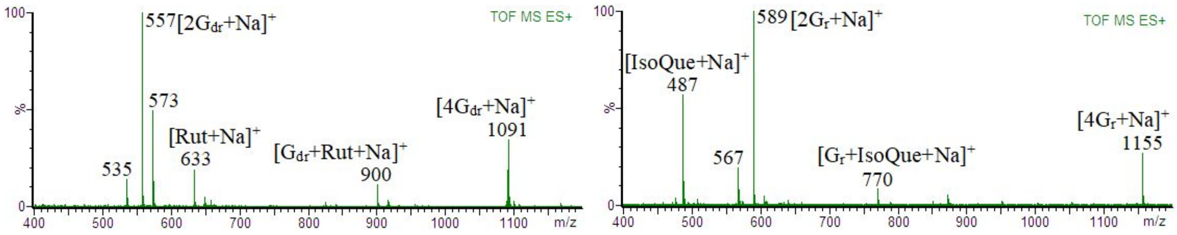

Figure S5. Exemplary full scan ESI mass spectra in the *m/z* range 400-1200.

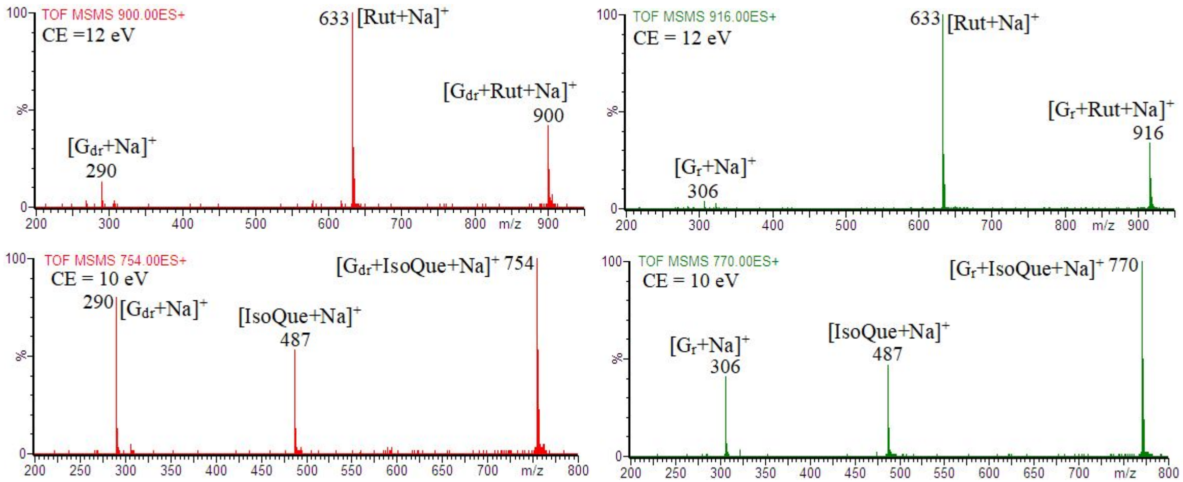

Figure S6. Exemplary product ion spectra of the $[G+M+Na]^+$ ions (M stands for isoflavone glycoside molecule). Product ion $[Rut+Na]^+$ is much more abundant than product ions $[G+Na]^+$, thus rutin has much higher Na^+ affinity than guanosine/deoxyguanosine. Product ion $[IsoQue+Na]^+$ has abundance comparable to the product ions $[G+Na]^+$, thus isoquercetin has similar Na^+ affinity as guanosine/deoxyguanosine.

Since the Na^+ affinity of flavonols is low, flavonols did not yielded the $[G+M+Na]^+$ ions.

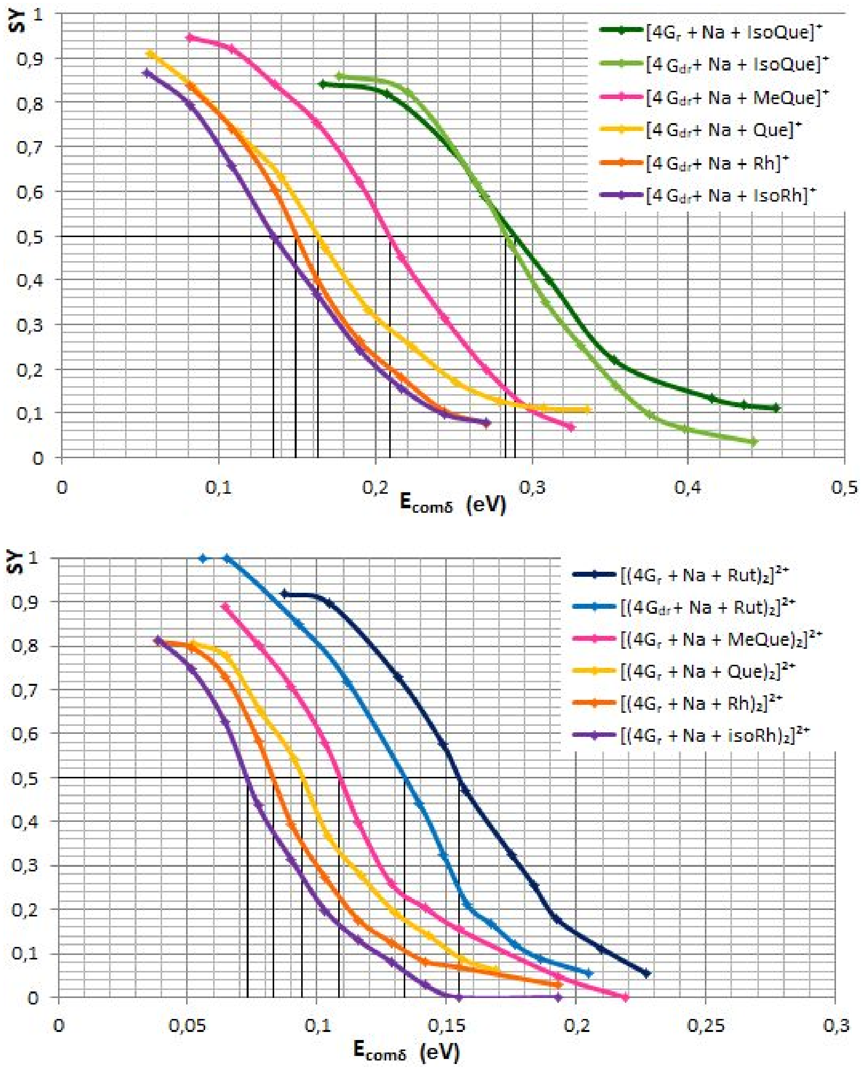

Figure S7. The obtained plots of SY against E_{com}□ for 1:1 and 2:2 adducts.

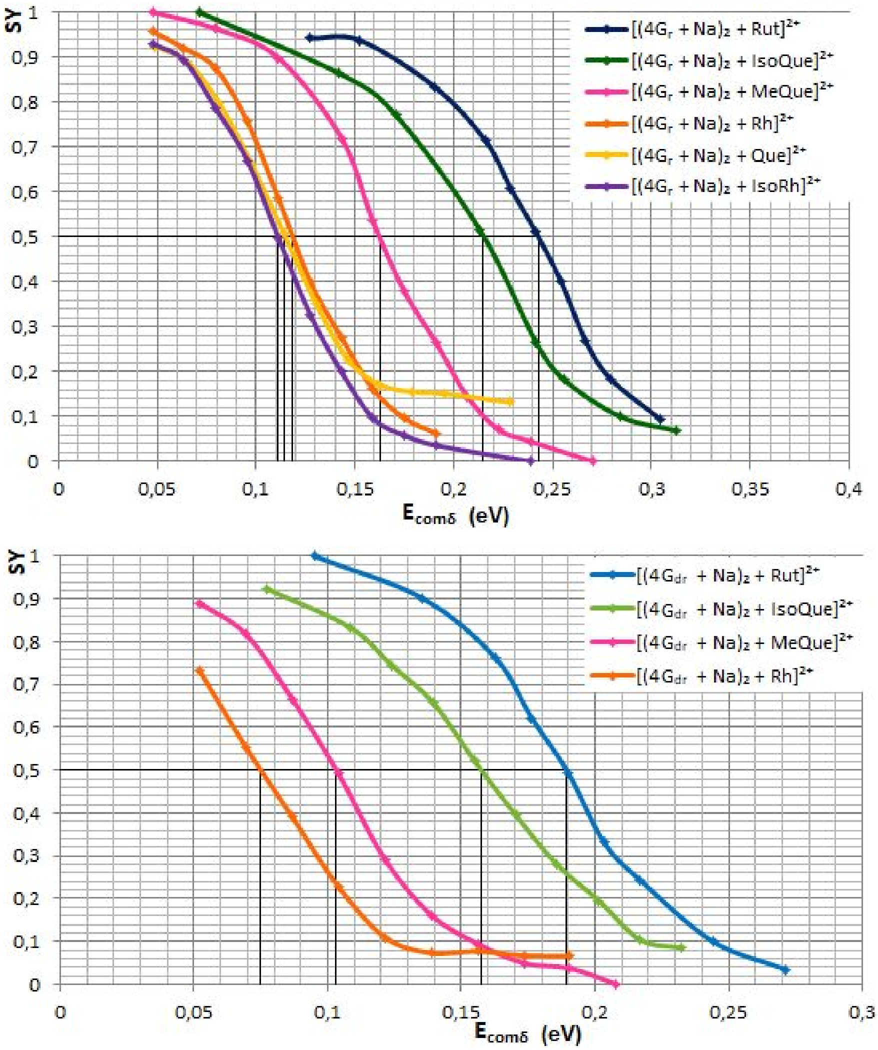

Figure S8. The obtained plots of SY against E_{com}□ for 2:1 adducts.
